# Unveiling and Characterizing Early Bilateral Interactions between Biofilm and the Mouse Innate Immune System

**DOI:** 10.3389/fmicb.2017.02309

**Published:** 2017-11-21

**Authors:** Christiane Forestier, Elisabeth Billard, Geneviève Milon, Pascale Gueirard

**Affiliations:** ^1^CNRS UMR 6023, Laboratoire Microorganismes: Génome et Environnement, Université Clermont-Auvergne, Clermont-Ferrand, France; ^2^INRA USC 2018, Inserm U1071, Laboratoire Microbes Intestin Inflammation et Susceptibilité de l’Hôte, Université Clermont-Auvergne, Clermont-Ferrand, France; ^3^Institut Pasteur, Paris, France

**Keywords:** bacteria, biofilm, intravital imaging, macrophage/monocyte, mouse, polymorphonuclear neutrophil

## Abstract

A very substantial progress has been made in our understanding of infectious diseases caused by invasive bacteria. Under their planktonic forms, bacteria transiently reside in the otherwise sterile mammal body tissues, as the physiological inflammation insures both their clearance and repair of any tissue damage. Yet, the bacteria prone to experience planktonic to biofilm developmental transition still need to be studied. Of note, sessile bacteria not only persist but also concur preventing the effectors and regulators of the physiological inflammation to operate. Thus, it is urgent to design biologically sound experimental approaches aimed to extract, at the earliest stage, immune signatures of mono-bacteria planktonic to biofilm developmental transition *in vivo* and *ex vivo*. Indeed, the transition is often the first event to which succeeds the “chronicization” process whereby classical bacteria-targeting therapies are no more efficacious. An *in vivo* model of micro-injection of *Staphylococcus aureus* planktonic or biofilm cells in the ear pinna dermis of laboratory transgenic mice with fluorescent immune cells is proposed. It allows visualizing, in real time, the range of the early interactions between the *S. aureus* and myeloid cell subsets- the resident macrophages and dendritic cells, the recruited neutrophil granulocytes/polymorphonuclear neutrophils, monocytes otherwise known to differentiate as macrophages or dendritic cells. One main objective is to extract contrasting immune signatures of the modulation of the physiological inflammation with respect to the two bacterial lifestyles.

## Introduction

Most invasive bacteria display two different lifestyles: whereas the free-floating planktonic bacteria’ life style dominates, in some clinical settings, bacteria sensing hostile conditions adhere to biotic or abiotic surfaces and form biofilms (Moormeier and Bayles, 2017). The planktonic to biofilm/sessile lifestyle transition is associated with important metabolic changes and self-production of proteins-lipids-exopolysaccharides-rich as well as extracellular DNA-containing extracellular matrix (Costerton et al., 1999).

According to the National Institutes of Health, biofilms have an enormous impact on human medicine, accounting for over 80% of infectious processes in otherwise sterile tissue (s). Whereas the physiological inflammation is able to both clear invasive planktonic bacteria and to repair tissue damages insuring the return to tissue structural and functional homeostasis, this physiological inflammation does not operate in tissues experiencing sustained colonization by bacterial biofilms. Moreover, contrasting with planktonic bacteria that are cleared by commonly used antibiotics, provided that they do not harbor genetic resistance determinants, the majority of bacteria within the biofilms are resistant to these antibiotics (Lebeaux et al., 2014).

In this review, our present understanding of the professional phagocyte sensors of microbial agonists expected to operate over the *in vivo* planktonic to biofilm lifestyle switch is briefly introduced. Transiently invasive planktonic bacteria are usually cleared by myeloid cells of either the neutrophil granulocyte lineage or/and by mononuclear phagocytes. However, the bilateral interactions engaged or not between sessile bacteria and the myeloid cells are still poorly studied. Until now, most experimentalists have conducted *in vitro* studies with sessile bacteria, exposing them to either one or the other myeloid cell lineage or both (Watters et al., 2016). This review focuses on recent developments obtained in rodent models to characterize inflammatory responses against *Staphylococcus aureus* or *Pseudomonas aeruginosa* sessile bacteria. A new experimental approach combining the mouse ear pinna model and the intravital imaging approach is proposed to analyze these innate immune responses at the dynamic level.

## The Professional Phagocyte Sensors of Microbial Agonists Expected to Operate Over the *In Vivo* Planktonic to Biofilm Lifestyle Switch

The *in vivo* developmental transition from planktonic to sessile bacteria reflects a range of bilateral cross talks in the fluctuating dynamic tissue milieu colonized by the bacteria under study. At the earliest stage of this developmental transition, communications between key bacterial messengers as well as interactions between bacterial agonists and sensors displayed by the resident and recruited myeloid cells such as the professional phagocytes are initiated and renewed.

### The Nucleotide-Based Second Messengers

The cyclic dinucleotides (c-di-NMPs) are recognized as Microbial Associated Molecular Patterns/MAMPs and induce a host type I interferon immune response prolonged by IFNγ production (Valle et al., 2013; Snyder et al., 2017). Moreover, the c-di-NMPs play a central role in many bacterial species during the lifestyle transition (Valle et al., 2013). Using the *P. aeruginosa* model organism, Valentini and Filloux (2016) showed that the bacteria use c-di-GMP as a checkpoint during the different steps of biofilm development. There is indeed a direct correlation between high levels of c-di-GMP in the bacteria and biofilm formation, and between low levels of c-di-GMP and motility (planktonic phenotype). The c-di-GMP second messenger is used by *Escherichia coli* and *Salmonella enterica* serovar Typhimurium over their planktonic to biofilm developmental transition (Allewell, 2016; Valentini and Filloux, 2016) whereas the c-di-AMP is used as a second messenger by other bacteria such as *S. aureus* (Corrigan et al., 2011).

The Guanosine tetraphosphate (ppGpp) and pentaphosphate (pppGpp), also called (p)ppGpp or alarmones are synthetized when bacteria are exposed to cells such as phagocytes in the infected tissue. Considered as bacterial signature of a so called stringent response, these alarmones represent intracellular signaling molecules known to participate to intracellular bacteria survival: Geiger et al. (2012) demonstrated that the stringent response is induced, *in vitro*, after *S. aureus* phagocytosis by neutrophil granulocytes or polymorphonuclears (PMN). The rapid (p)ppGpp synthesis leads to increased *psm* transcription and to participation of synthetized phenol soluble modulins (PSMs) concurring to bacteria survival after phagocytosis (Geiger et al., 2012). Depicted as pro-inflammatory agents, the PSMs also account for the bacteria escape from the transient intracellular niche, followed either by bacteria survival inside the cytosol or by lysis of the cell, all these rapid processes contributing to damages of the *S. aureus-* hosting tissues (Geiger et al., 2012; Peschel and Otto, 2013).

### The Quorum Sensing Circuit and Its Additional Regulators

Quorum sensing (QS) is a cell-to-cell signaling process that allows bacteria to sense and process high cell densities. It involves the synthesis, release and accumulation of signaling molecules called auto-inducers (AIs) (Papenfort and Bassler, 2016). At high concentrations, AIs induce cellular signaling cascades that notably control biofilm formation. The QS system of *S. aureus* called accessory gene regulator (Agr) has been extensively studied (Paharik and Horswill, 2016). With other regulators, it constitutes a complex regulatory network that, at any moment, either modifies AGR activity itself, or its downstream signaling or metabolic pathways. At the early stage of *S. aureus* developmental transition from planktonic to biofilm lifestyle, the low Agr concentration allows the production of inter-bacteria/intercellular adhesins whereas toxins’ expression is repressed (Balasubramanian et al., 2016). Later, high levels of Agr induce biofilm structuration and dispersion by up-regulating the expression of the pro-inflammatory PSM molecules (Otto, 2008; Periasamy et al., 2012; Kavanaugh and Horswill, 2016; Paharik and Horswill, 2016). Environmental factors also modulate the Agr function in *S. aureus*, with a well-described inhibitory effect of the reactive oxygen species (ROS) produced by innate immune cells (Kavanaugh and Horswill, 2016).

Among other regulators that interact with the *agr* system at later stage of the biofilm development and maturation, the *S. aureus* exoprotein (Sae) two-component system promotes the synthesis and secretion of leucocidins and other virulence determinants that actively participate to *S. aureus* survival after contact with PMN *in vitro* (Paharik and Horswill, 2016). Sae mutants have indeed a decreased capacity to resist to the PMN- clearing functions after contact (Yarwood and Schlievert, 2003; Voyich et al., 2009; Paharik and Horswill, 2016).

## Rodent Models to Study Inflammatory Responses to Biofilms *In Vivo*

In rodent laboratory models, the most rapidly recruited cells are phagocytes, namely the PMN. Over any local disruption of mouse tissue homeostasis, PMN stored in the bone marrow egress into the blood vascular bed. Through chemoattractants and cytokines produced by tissue resident mast cells and macrophages (Teng et al., 2017), the PMN rapidly cross the microvessels endothelial cells, reaching the extracellular compartment colonized by invasive bacteria. They represent the first wave of innate immune cells to be recruited from the blood circulation. Most often, PMN efficiently kill and degrade invasive planktonic bacteria by using different antimicrobial strategies: phagocytosis, production of ROS and antimicrobial peptides, as well as cytotoxic components released from their subcellular distinct granules. More recently, neutrophil extracellular traps (NETs) have been observed and included as an additional strategy (Brinkmann et al., 2004). Among the *in vivo* models that allow characterization of the bilateral interactions co-engaged by PMN and sessile bacteria, we selected those relying on either *P. aeruginosa* or *S. aureus* (**Table 1**).

**Table 1:** Rodent models to study inflammatory responses to biofilms *in vivo*.

Organism	Biomedical device whenever it is implanted	Tissue(s) where are either inoculated bacteria or implanted a bacteria–free or loaded device	Either bacteria inoculation or bacteria-loaded device delivery mode	Inoculum dose (CFU)	Reference
*P. aeruginosa*	–	Lung (immobilized bacteria in alginate beads)	Intra-tracheal	6 × 10^6^ to 1,5 × 10^7^	Jensen et al., 2004; Bjarnsholt et al., 2005; Alhede et al., 2009; van Gennip et al., 2009; Jakobsen et al., 2012
	–	Oropharyngeal aspiration of a bacterial suspension	Oropharyngeal	1,5 × 10^7^	Secor et al., 2017
	Biofilm pre-colonized silicone implant	Implant inserted into the peritoneal cavity (hollow tubes)	Intraperitoneal	–	Van Gennip et al., 2012
	Biofilm pre-colonized silicone implant	Implant inserted into the peritoneal cavity (flat implant)	Intraperitoneal	–	Christensen et al., 2007
*S. aureus*	–	Diabetic mouse-based model allowing systemic invasion outcome	Intraperitoneal	10^8^	Hanses et al., 2011
*P. aeruginosa* and *S. aureus*	–	Chronically wounded diabetic mouse model	Topical application of bacteria onto a peripheral wound	10^4^ 2 × 10^5^, 2 × 10^6^, 2 × 10^7^	Watters et al., 2013, 2014 Guo et al., 2013
*S. aureus*	Un-colonized silicon splint	Chronically wounded diabetic mouse model	Dermal application of bacteria onto the wounded surface	10^6^	Nguyen et al., 2013
*P. aeruginosa*	–	Chronic wound model	Subcutaneous injection of bacteria immobilized in alginate beads beneath a thermal skin lesion	10^6^	Trøstrup et al., 2013
*S. aureus*	–	Air pouch model	Inoculation of bacteria in a single pouch	10^7^	Torre et al., 2015
*S. aureus*	–	Hematogenous model of septic arthritis initiated in murine knees	Intravenous	2 × 10^6^ 1,5 × 10^6^, 3 × 10^7^	Corrado et al., 2016 Verdrengh and Tarkowski, 1997
	Un-colonized orthopedic implant (K-wire)	Post-arthroplasty model (K-wire into the right-knee joint)	Inoculation of bacteria into the joint space containing the cut end of the implant	10^3^ 10^2^, 10^3^, or 10^4^10^3^10^3^ or 10^5^	Bernthal et al., 2011; Heim et al., 2014; Scherr et al., 2015 Pribaz et al., 2012 Niska et al., 2012 Vidlak and Kielian, 2016
	Un-colonized catheter inserted in a pouch	Catheter-related model	Intradermal inoculation of bacteria at proximity of the pouch containing the catheter	5 × 10^5^	Silva-Santana et al., 2016
	Un-colonized catheter	Catheter-related model	Intradermal inoculation of bacteria into the catheter lumen	10^3^5 × 10^5^	Hanke et al., 2012, 2013 Thurlow et al., 2011
	Un-colonized orthopedic implant (K-wire)	Hematogenous implant-related bacteria colonization	Intravenous (tail vein) Intravenous (retroorbital sinus)	10^4^ to 10^9^ 10^6^, 5 × 10^6^, 10^7^	Shiels et al., 2015 Wang et al., 2017
	Pre-colonized flat stainless steel wire	Orthopedic biofilm model	Tibial implant	–	Nishitani et al., 2015
	Pre-colonized pin	Prosthetic implant model	Tibial implant	3 × 10^5^	Prabhakara et al., 2011a,b
*P. aeruginosa*	Un-colonized polyethylene catheter	Ascending pyelonephritis model	Inoculation of bacteria into the bladder	5 × 10^6^	Mittal et al., 2009

Of note, the features of the biofilm-colonized devices condition the interaction profiles: whereas in devices of hollow type (catheter, silicon splint) bacterial biofilms are protected from the immune cells, on solid devices (K-wire, pin), the bacterial biofilms are directly exposed to the different waves of immune cells. Depending on the model used, many other parameters operate (listed in **Table 1**). In most models, an intense and rapid accumulation of PMN is observed at the proximity of the biofilms (Wagner et al., 2003; Prabhakara et al., 2011a; Torre et al., 2015; Moser et al., 2017; Wang et al., 2017), the PMN representing the most abundant population of recruited cells. Using a non- invasive bioluminescence-based approach, Bernthal et al. (2011) showed that this recruitment is IL-1β dependent, as a 50% decrease in PMNs numbers was observed in the bacteria colonized knee joints of IL-1β deficient mice, as compared to wild-type mice. This PMN infiltration is increased when diabetic mice are treated with insulin in a bacteria-hosting wound, the latter incorporating actin and DNA from lysed PMN, which therefore contributes to the building of biofilms (Watters et al., 2014). Of note, a low PMN recruitment in the target tissues (Thurlow et al., 2011; Scherr et al., 2014; Secor et al., 2017) can be also operating: in particular was noticed an association between the reduced PMN recruitment and the production of Filamentous Pf1-like bacteriophage (Pf phage) by *P. aeruginosa* (Secor et al., 2017). Taken globally, whatever the experimental conditions depicted in **Table 1**, there is a need to capture more comprehensive information documenting, at least at the earliest stages, the *in vivo* dynamic interactions between bacteria and PMN.

Could mature biofilms’ matrix components protect bacteria from activated PMN? PMN were indeed shown to be activated, *in vitro*, by bacterial DNA and polysaccharides components such as alginate, the measured outcome being an increase of their respiratory burst (Pedersen et al., 1990; Alvarez et al., 2006; Fuxman Bass et al., 2008; Jensen et al., 2010). *In vivo, P. aeruginosa* biofilm interactions with PMN lead to up regulation of the QS-dependent effectors such as rhamnolipids which cause PMN lysis (Alhede et al., 2014). This shielding specific property of rhamnolipids is described in mouse models relying upon the intraperitoneal implant of pre-colonized silicone device (Van Gennip et al., 2012) or on biofilm development in the respiratory tract (Bjarnsholt et al., 2005; Jakobsen et al., 2012). In these models, QS mutants are unable to produce rhamnolipids and are rapidly phagocytosed and cleared by PMN.

Monocytes/macrophages are other key actors recruited and sensing both the other inflammatory cells -e.g., PMN -as well as bacteria agonists. Under homeostatic conditions, circulating macrophage/monocytes qualified as classical/intermediate ones are continuously recruited from the blood and either mature into macrophages or remain as monocytes within tissues (Sprangers et al., 2016; Jakubzick et al., 2017). In the skin, long- lived resident macrophages are also present and maintain their population by self-renewal (Sprangers et al., 2016; Jakubzick et al., 2017). As for PMN, microbe-specific molecules participate to the rapid emigration of classical/intermediate monocytes in the extravascular space which contribute to the resolution of inflammation by recognizing and phagocytosing bacteria and dying cells, and by producing ROS and reactive nitrogen species (RNI) (Sprangers et al., 2016; Jakubzick et al., 2017; Kashem et al., 2017). Once PMNs experience apoptotic death, a second wave of monocytes qualified as non- classical monocytes contribute to the resolution of inflammation and repair of the disrupted tissue (Sprangers et al., 2016).

Only a few studies (Prabhakara et al., 2011a; Thurlow et al., 2011; Hanke et al., 2012, 2013; Scherr et al., 2015; Corrado et al., 2016; Silva-Santana et al., 2016; see **Table 1**) assess the complex recruitment waves and networks of both PMN and monocyte subsets. Of note, an early wave of monocyte recruitment is sometimes predominant, as compared to PMN recruitment (Thurlow et al., 2011). Two main studies were conducted to monitor the abundance and functional features of monocyte/macrophage recruitment in *vivo* at specific time points after contact with biofilms. The biofilms’ matrix components clearly modulate the functional properties of recruited cells. In a *S. aureus* pre-colonized catheter-based model, mobilized cells are mainly distant from the catheter and a non-significant proportion of F4/80^+^ macrophages are able to invade the biofilms. Cells present deeply into the biofilm rapidly die, therefore preventing phagocytosis of biofilm bacteria to be otherwise exerted by macrophages (Thurlow et al., 2011). A significant reduction of pro-inflammatory cytokines (IL-1β, TNF…) and chemokines (CXCL2, CCL2) production at the boundary of the biofilm- colonized tissue is also observed, associated with a reduced Nitric Oxide synthase (iNOS) induction and an increased arginase-1 expression. The authors conclude to a macrophage polarization toward a counter-inflammatory activated M2 phenotype and to fibrosis with MyD88- signaling as a major effector pathway regulating these two phenomena (Thurlow et al., 2011; Hanke and Kielian, 2012; Hanke et al., 2012). In a lung experiencing colonization with phage Pf- producing *P. aeruginosa*, Secor et al. (2017) also documented a M2 polarization profile. Taken globally, it appears that the macrophage M2 polarization and fibrosis do concur prolonging bacteria biofilm persistence, although the universal character of such responses remains to be assessed.

## The Mouse Ear Pinna Dermis Imaged By Intra-Vital Confocal Microscopy At Steady and Not Steady State

Intra-vital microscopy is increasingly used in different biomedical research fields to study dynamic processes at the cellular level in their specific tissue environment. Compared to classical methods such as *ex vivo* histology or flow cytometry, intra-vital confocal microscopy live imaging allows dynamic interactions to be captured once fluorescent reporters are expressed by both the microbes and the laboratory mouse cell lineages with which are engaged, more or less durably, dynamic interactions.

Several skin-related models were described in the literature to study the immunobiology of biofilm infections by using classical approaches (**Table 1**). In these models, the biofilm-loaded cutaneous sites were the back or the flank of animals, which represent unsuitable sites for intravital microscopy of cutaneous innate immune responses.

The ear pinnae is one of the most studied appendage in which are delivered microorganisms, enabling the observation of the early and either transient or prolonged dynamic interactions with resident or recruited myeloid cells (Amino et al., 2006, 2007; Peters et al., 2008; Ng et al., 2011; Sumaria et al., 2011; Jain and Weninger, 2013; Tavares et al., 2013; Carneiro et al., 2017). As an imaging site, the ear presents several technical advantages such as the accessibility of the tissue, easy and fast protocols of preparation to perform imaging experiments and obtain reproducible results, and the possibility of imaging for long periods of time in blocks of 20–40 min (Li et al., 2012).

The mouse ear pinna appendage harbors a thin epidermis and an underlying dermis, respectively avascularized and highly vascularized, which contain a broad range of lympho-myeloid cells (Jain and Weninger, 2013). Using a multiphoton microscopy approach and quantitative flow cytometry, Tong et al. (2015) elaborated a 3D immune cell atlas of mouse skin and compared the ear pinnae, dorsal back, footpad, and tail skin. Langerhans cells and dendritic epidermal T cells are present in the epidermis, whereas the dermis mainly harbors in the upper dermis myeloid cells such as dendritic cells, mast cells as well as lymphoid cells (αβT cells, γδT cells, and group 2 Innate Lymphoid Cells), and mainly resident macrophages in the deeper dermis (Tay et al., 2014; Tong et al., 2015). All these dermis-located immune cells are included in a collagen- and elastin- rich and more or less hyaluronan- rich extracellular matrix. In term of cell numbers, the specificities of the ear pinna cutaneous site are the following ones: the total leukocyte density is high, with around 4800 cells per mm^2^ and a majority of cells present in the dermis. Macrophages, dermal dendritic cells and mast cells represent the majority of ear dermal leukocytes with respectively around 6000 macrophages per mm^3^ and more than 2000 dermal dendritic cells and mast cells per mm^3^ (Tong et al., 2015). Of note, the ear pinna dermis presents two specificities regarding the presence of mast cells: their high prevalence and their perivascular localization, in close association to blood vessels, in contrast to dermal dendritic cells (Tong et al., 2015). By using intravital multi-photon microscopy, Ng et al. (2011) showed that PMNs are present in the ear dermis of naive mice, but in small numbers, and patrol, as do dermal dendritic cells. Both cell lineages are likely surveying the presence of either epidermis-restricted microbiota derived agonists that reach the dermis or endogenous agonists through the sensors displayed at the plasma membrane or within the macropinocytosis/endosomal machinery.

## The Mouse Ear Skin Model to Study the Dynamics of Innate Immune Responses Against Planktonic or Sessile *Staphylococcus aureus*

**FIGURE 1:**
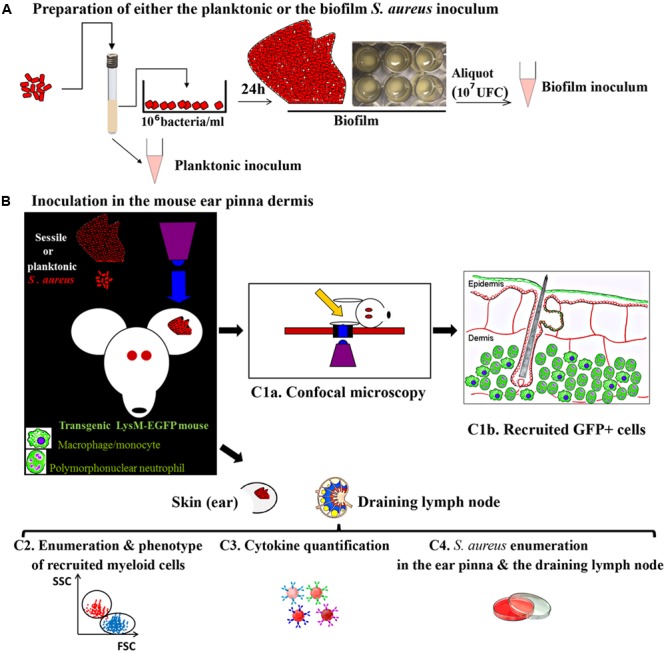
The three steps’ workflow proposed for extracting, post either *Staphylococcus aureus* planktonic or biofilm delivery, specific immune signatures displayed by mouse myeloid cells. **(A)** Inoculum preparation *in vitro*. **(B)** Inoculum micro-injection in the ear pinna of mouse (10^7^ UFC of either planktonic or sessile bacteria). (C1a) Real time imaging of the ear pinna of a LysM-EGFP transgenic mouse harboring fluorescent phagocytes and fluorescent *S. aureus* bacteria by confocal microscopy. (C1b) Massive recruitment of GFP+ cells post-injection in the upper dermis of the ear pinna of a mouse inoculated with *S. aureus* sessile or planktonic bacteria. (C2), (C3), (C4) Quantitative analyses in the ear pinna and the auricular lymph node to determine (C2) the phenotype of recruited cells, (C3) the cytokine levels and (C4) the bacteria counts.

By combining intra-vital confocal microscopy approach and the mouse ear pinna infection model, inflammatory responses against biofilms could be analyzed for the first time at the dynamic level in the tissue environment. The **Figure 1** shows the schematic workflow of methods proposed to characterize and compare the innate immune responses against *S. aureus* planktonic and sessile bacteria. Transgenic mice with fluorescent immune cells visible in the skin such as Lysozyme-EGFP (circulating PMNs and monocytes, dermal macrophages), CD11c-EYFP (dermal dendritic cells, epidermal langherans cells) and Mcpt5-Cre+R26Y+ (dermal mast cells) mouse strains have been selected (Faust et al., 2000; Lindquist et al., 2004; Dudeck et al., 2011). The conditions to prepare bacteria inoculum will be set up. The planktonic inoculum will be obtained from an overnight culture of fluorescent *S. aureus* Lyo-S2 strain in Trypticase Soja broth (Marquès et al., 2015). Biofilms will be generated from a planktonic culture incubated at 37°C under static conditions. After 24 h of incubation, biofilms will be gently collected (A). The first series of experiments will be performed with Lysozyme-EGFP mice inoculated into the ear tissue with the same number of CFU of either planktonic or sessile bacteria (B). Inoculum will be micro-injected in two injection points in the dermis of the ear pinna tissue with a nanofil syringe (Mac-Daniel et al., 2016). At early time-points, mice will be anesthetized and the cellular recruitment will be analyzed by confocal microscopy at the injection points (C1a and C1b). The behavior of recruited or resident innate immune cells (moving speed, trajectory, distance covered) will be analyzed, as well as their specific interactions with bacteria. In parallel, additional groups of mice will be inoculated with planktonic or biofilm bacteria to perform quantitative analyzes over 14 days in the ear tissue and the cutaneous draining lymph node (auricular lymph node), i.e., determination of the phenotype of recruited cells (C2), of the cytokine levels (C3), and counting of bacteria in the target tissues (C4). The inflammation visible to the eye and macroscopic cutaneous lesions (skin necrosis) will be observed and compared.

## Conclusion

The objective of the review was to highlight the requirement of developing new *in vivo* models to analyze, at the early stage of infection, the dynamics of innate immune responses against *S. aureus* planktonic or sessile bacteria. A new experimental approach in the field combining the mouse ear pinna model and the intravital imaging approach is proposed. The long term objectives are to use this model as a pre-clinical model to test necessary new therapeutic approaches targeting the host immune system, as proposed initially by Hanke et al. (2013).

## Author Contributions

CF: intellectual contribution to the work; EB: intellectual contribution to the work; GM: substantial, direct and intellectual contribution to the work; PG: substantial, direct and major intellectual contribution to the work-corresponding author. All authors listed approved the work for publication.

## Conflict of Interest Statement

The authors declare that the research was conducted in the absence of any commercial or financial relationships that could be construed as a potential conflict of interest.
